# An Overview on the Role of Ionic Liquids and Deep Eutectic Solvents in Oral Pharmaceuticals

**DOI:** 10.3390/pharmaceutics17030300

**Published:** 2025-02-25

**Authors:** Stefano Sangiorgi, Beatrice Albertini, Serena Bertoni, Nadia Passerini

**Affiliations:** Department of Pharmacy and Biotechnology, University of Bologna, Via San Donato 19/2, I-40127 Bologna, Italy; stefano.sangiorgi9@unibo.it (S.S.); serena.bertoni4@unibo.it (S.B.); nadia.passerini@unibo.it (N.P.)

**Keywords:** ILs, DESs, API-ILs, NaDES, manufacturing methods, toxicity, oral drug delivery, drug solubilization, bioavailability enhancement, green chemistry

## Abstract

Over the past twenty years, ionic liquids (ILs) and deep eutectic solvents (DESs) have gained recognition across various fields, including catalysis, extraction and purification, materials science, and biotechnology. Notably, the use of ILs and DESs in pharmaceutical research, especially in drug delivery, has seen remarkable expansion over the past decade. This review offers a comprehensive analysis of ILs and DESs specifically designed for the oral administration of drugs having unfavorable biopharmaceutical properties. The classification and characteristics of ILs and DESs, along with their newer natural (Bio-ILs and NaDESs) and therapeutic subcategories (API-ILs and TheDESs) are outlined. Additionally, a further subgroup of ILs, known as surface active ionic liquids (SAILs), is described. Then, a detailed examination of the available manufacturing methods in a sustainable, time-consuming, and scalable perspective, and toxicity concerns in relation to their subdivision are evaluated. Finally, their specific applications in oral drug delivery, whether used as neat solvents or converted into administrable dosage forms, are analyzed and discussed. Despite the significant advancements in recent years regarding the use of these solvents in oral drug delivery, there are still many aspects that need further investigation. These include their interaction with biological systems (gastrointestinal fluids and mucosa), their long-term stability, and the development of effective drug delivery systems.

## 1. Challenges in Oral Drug Delivery

**Figure 1:**
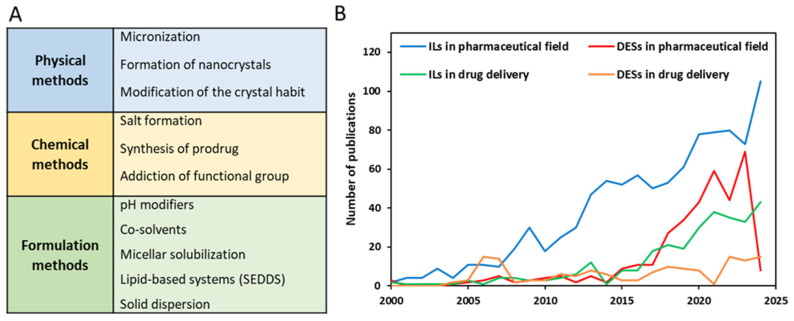
(**A**) Main techniques used for the bioavailability enhancement of poorly soluble APIs. (**B**) Number of publications on ILs (PubMed (https://pubmed.ncbi.nlm.nih.gov/?term=ILs+pharmaceuticals&filter=years.2000-2025&sort=fauth); (https://pubmed.ncbi.nlm.nih.gov/?term=ILs+drug+delivery&filter=years.2000-2025&sort=fauth) accessed on 8 January 2025) and DESs (PubMed (https://pubmed.ncbi.nlm.nih.gov/?term=DESs+pharmaceuticals&filter=years.2000-2025&sort=fauth); (https://pubmed.ncbi.nlm.nih.gov/?term=DESs+drug+delivery&sort=fauth) accessed on 8 January 2025) in pharmaceutical fields and in drug delivery from 2020 to the present.

Current estimates suggest that oral formulations account for approximately 90% of the global market share of all pharmaceutical formulations intended for human use [1]. This dominance is attributed to factors like high patient compliance, convenient handling, flexibility in dosage forms, cost-effectiveness, and the ease of large-scale manufacturing [2]. However, the oral bioavailability of drugs is still a challenging concern. The Biopharmaceutics Classification System (BCS) is a valuable tool in drug formulation development, categorizing orally administered APIs into four classes based on their aqueous solubility across the gastrointestinal (GI) pH range and permeability across the GI mucosa in relation to their dose. Nowadays, poorly water-soluble drugs, belonging to class II (low solubility/high permeability) and IV (low solubility/low permeability) of the BCS, constitute the 80% of new drug candidates and approximately 40% of the marketed oral drugs that are categorized as practically insoluble (<100 μg/mL) [3]. Poor oral bioavailability leads to reduced drug efficacy and often requires the administration of high doses with a greater risk of side effects, representing the major causes of drug failure in preclinical and clinical development [1]. For all of these reasons, the design and development of innovative and effective oral formulations have emerged as one of the primary challenges for the pharmaceutical industry in recent years. Since the early 2020s, numerous methods have been documented in the literature to improve drug solubilization and absorption. These methods encompass physical, chemical, and various formulation techniques, employing both bottom-up and top-down technologies, as outlined in Figure 1A. Each of these methods presents advantages and disadvantages. The main challenges often involve chemical and physical stability, limited drug loading capacity, multiple manufacturing steps that require organic solvents, high production costs, inconsistent batch-to-batch reproducibility, and mostly, health and environmental concerns [4].

From a sustainable perspective, it would be far more advantageous to use solvents that can enhance the biopharmaceutical properties of therapeutic molecules, thus promoting the production of bio-enabling drug delivery systems. Therefore, given the growing concern regarding the use of traditional solvents, new categories of promising solvents offer environmentally friendly alternatives to traditional solvents contributing to greener chemistry and more sustainable industrial practices. These solvents, defined as ionic liquids (ILs) and deep eutectic solvents (DESs) [5], have emerged in different fields such as catalysis, electrochemistry, extraction and purification, green chemistry, material science, pharmaceuticals, and biotechnology due to their unique properties. Figure 1B demonstrates the rapid growth in publications on ILs and DESs within the pharmaceutical field, particularly in drug delivery.

This review provides a thorough examination of ILs and DESs designed for oral administration, a route that has been less explored to date compared to transdermal delivery. Initially, a brief description of the properties of ILs and DESs, along with their classification into various subcategories, is provided. The subsequent sections focus on ILs and DESs that are potentially suitable for oral administration, evaluating aspects such as the scalability and sustainability of production methods, toxicity concerns, and their applications in oral drug delivery.

## 2. Ionic Liquids: Physicochemical Properties and Classification

**Figure 2:**
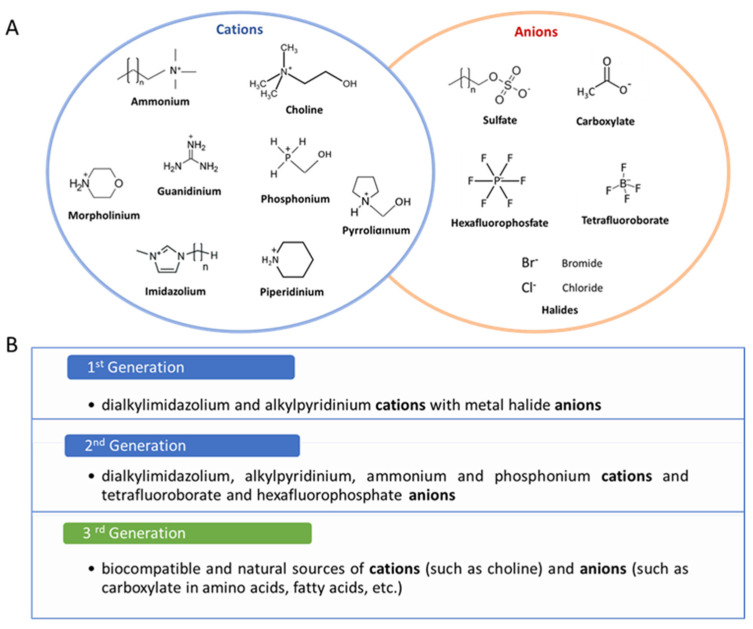
(**A**) Common cations and anions used for the preparation of ILs and (**B**) ILs classification.

In 1914, Paul Walden reported the possibility of having salts, by a simple acid–base neutralization reaction, that remain liquid at room temperature, which are nowadays known as ionic liquids (ILs). ILs are also known as ionic fluids, ionic melts, liquid electrolytes, liquid salts, ionic glasses, and designer solvents. ILs found their first application in synthesis, catalysis, extraction, and electrochemistry. In the pharmaceutical field, ILs are recognized as excellent solvents and cosolvents for small molecule drugs and good permeation enhancers, allowing their application in topical, transdermal, and oral drug delivery [6,7]. Typically, ILs are molten organic salts composed of asymmetrical organic cations paired with inorganic or organic anions, characterized by a melting point ≤ 100 °C and with extremely low vapor pressure. They have emerged as alternatives to organic solvents, and in comparison, to the approximately 600 molecular solvents currently in use, there are at least 1 million binary ILs and 1018 ternary ILs [8]. At the beginning, ILs have received little attention, because of their instability at room temperature, especially those having halide anions. However, since Wilkes and Zaworotko reported the first imidazolium-based IL that was stable in air and water in 1992 [9], the literature on ILs has increased year by year (Figure 1B). ILs exhibit significant versatility because their chemical properties can be easily adjusted by altering the structures of their cations and anions. For instance, the viscosity and density of ILs can be controlled, as these characteristics are influenced by the ion structures [10,11]. Additionally, ILs can possess a broad spectrum of polarities, ranging from highly polar to non-polar, depending on the chosen cation and anion. Typically, anions are small and inorganic, whereas cations are larger and organic (Figure 2A). The disparity in ion sizes leads to decreased crystallinity in the system, enabling ILs to remain liquid at lower temperatures [11]. This flexibility allows the design of ILs with specific properties for a desired application.

Three generations of ILs exists, each suited for different applications based on their chemical structure and properties, and their classification is reported in Figure 2B. The first-generation ILs possess a low melting point, high thermal stability, low vapor pressure, and a broad range of fluidity. Despite these attractive physical properties, first-generation ILs are sensitive to water and air, non-biodegradable, and exhibit aquatic toxicity [12]. Additionally, ionic liquids with halides as the anionic part had negligible biodegradability due to the lack of a carbon source [13]. The second-generation ILs are stable in both air and water, and feature adjustable physical and chemical properties, but are characterized by high toxicity and poor biodegradability [14]. The third-generation ILs, which include biologically active ions, are particularly well-suited for biopharmaceutical applications. Compared with first- and second-generation ILs, these ILs offer several advantages, such as low toxicity, reduced manufacturing costs, and good biodegradability [15]. For instance, Bio-ILs are derived from cholinium, betainium, and cartinium, in contrast to conventional ILs, which are based on imidazolium and pyridinium [11]. The biodegradation of ILs is influenced by the length of the alkyl chain, with longer chains leading to increased biodegradation due to more oxidizable carbon atoms. The presence of an ester in the side chain of an ionic liquid enhances biodegradation, whereas amide derivatives exhibit poor biodegradability. These biocompatible ILs, referred to as Bio-ILs, are increasingly utilized in pharmaceutical formulation due to their benefits in solubility, formulation stability, and efficient delivery (e.g., via oral, topical, and transdermal routes) [16,17].

**Figure 3:**
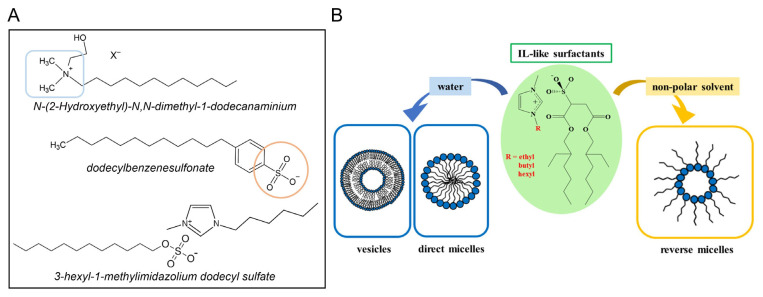
(**A**) Examples of cationic, anionic, and bi-amphiphilic surface active ILs (SAILs). (**B**) Schematic representation of the self-aggregation behavior of SAILs in water and non-polar solvents. Adapted with permission from Ref. [18]. Copyright 2020, American Chemical Society.

Surface active ionic liquids (SAILs) are ILs with surface activity and can be achieved by incorporating a long alkyl chain into the cation, anion, or both. As an example, the structure of these amphiphilic molecules is illustrated in Figure 3A. This modification allows the compound to exhibit behavior similar to conventional surfactants as well as comparable critical micelle concentration (CMC) values. This means that these compounds can self-assemble in an aqueous solution providing various colloidal systems, as illustrated in Figure 3B [13].

### API-Ionic Liquids

Active pharmaceutical ingredient-based ILs, namely API-ILs, are formed by pairing a basic or acidic API with an appropriate counterion; API-ILs may also include dual APIs with opposite charge [19]. API-ILs were first introduced in 2007 with the synthesis of ranitidine docusate [20]. API-ILs are a fascinating area of research due to their potential to enhance the solubility, thermal stability, and bioavailability of drugs. Issues related to polymorphism, commonly associated with solid dosage forms, can also be addressed by API-ILs [7], as interactions between the counterparts prevent the processes of nucleation and crystal growth of the precursors. Additionally, by selecting suitable counterions for parent APIs, it is possible to exert control over both their physicochemical and biological properties. The full range of API-IL counterions suitable for pharmaceutical use has yet to be fully cataloged. However, the most extensively studied counterions for API-IL synthesis include choline, docusate, quaternary ammonium, imidazolium, phosphonium, and amino acid-derived cations [7,21].

So far, three types of API-ILs have been identified based on their formation mechanisms. The most prevalent type is created directly through ionic binding, using APIs as either anions or cations. The second type involves covalent linkage, forming ionic prodrugs of neutral APIs, which must be synthesized via covalent bonds before being converted into ILs. For instance, neutral paracetamol was transformed into an ionizable form by pairing it with the docusate counterion, resulting in the corresponding IL [22]. The third type utilizes both methods to produce dual active API-ILs [19].

## 3. Deep Eutectic Solvents: Physicochemical Properties and Classification

**Figure 4:**
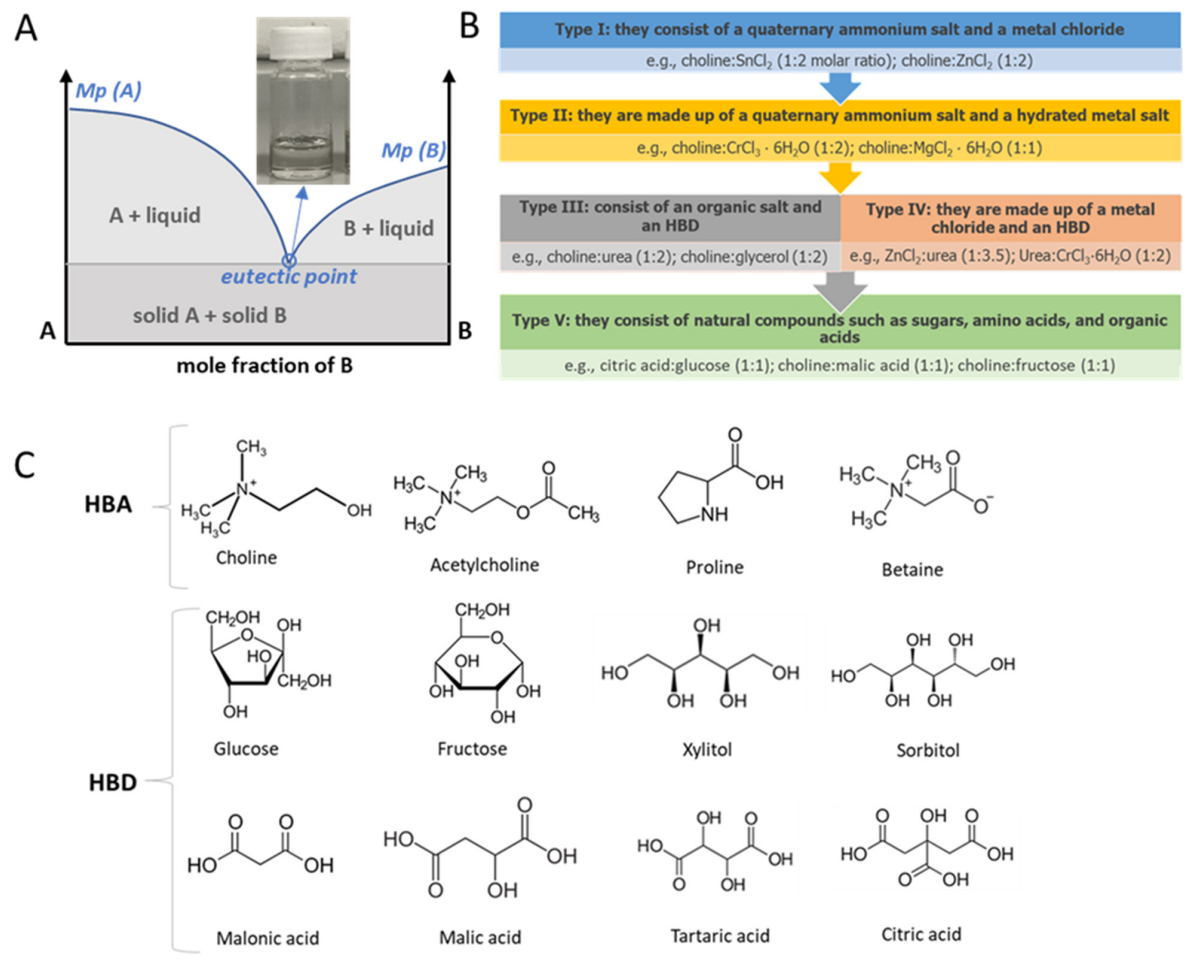
Schematic representation of (**A**) a eutectic point on a two-component phase diagram; (**B**) DES classification; and (**C**) common HBA and HBD constituents of NaDESs.

In 2003, deep eutectic solvents (DESs) were introduced by Abbott et al. [23] to describe mixtures of amides and quaternary ammonium salts with melting temperatures far below those of their pure components. DESs were initially classified as ILs due to their similarity in properties. Subsequent studies have highlighted how these two systems mainly differ in the type of involved interactions. ILs are, in fact, liquids composed of two charged species linked by an ionic bond in which the proton transfer is essential for salt formation, while DESs are solvents mainly characterized by the presence of hydrogen bonding. The difference in bonds may result in a greater toxicity of the former compared to the latter, making DESs particularly interesting for the pharmaceutical industry. DESs are obtained by the interaction between an appropriate amount of a hydrogen bond donor (HBD) and a hydrogen bond acceptor (HBA) [5], usually reported in specific molar ratios. The result is a binary eutectic mixture stable at room temperature characterized by a melting point significantly lower than that of the individual species (Figure 4A). During the formation of DESs, a network of hydrogen bonds is established, supported by Van der Waals forces and electrostatic repulsion, all of which contribute to lowering the melting point, allowing the mixture to remain in a liquid state at room temperature [24]. From a thermodynamic point of view, the constitution of these systems can be associated with an endothermic process and a positive enthalpy variation. Such eutectic mixtures, having lower lattice energy, are easily miscible in water reducing the risk of drug recrystallization [25]. Due to the hydrogen bond network, a certain amount of water can be added to aid in the formation of these bonds resulting in ternary systems. On the other hand, the appropriate percentage of water added should not exceed 30–40% as otherwise the hydrogen bond network would be destroyed [26].

The physicochemical properties of these solvents are crucial in determining the appropriate context for the use of a specific DES. Certainly, the primary characteristic to assess is the melting point. DESs typically have a lower melting point than their individual components, ideally around room temperature. This property is influenced by the bond strength between the HBA and HBD, the system’s entropy and factors such as the size and nature of the DES components [27]. Other important properties include the density, viscosity, and rheological behavior of these systems. Generally, DESs exhibit a much higher density compared to water and ILs, due to the molecular interactions within eutectic mixtures, which vary with temperature. The same goes for viscosity, as DESs tend to be more viscous than other solvents at room temperature. This property is strongly influenced by the molar ratios and by the number of carboxyl and hydroxyl groups which are responsible for forming a larger number of hydrogen bonds. In this regard, it is important to remember that excessive viscosity is a disadvantage for pharmaceutical development, as it could hinder handling and oral administration.

Smith and colleagues [28] categorized DESs into four types (Types I–IV) based on their composition and chemical structure. However, some newly developed DESs did not fit into these categories, leading to the introduction of a fifth type, Type V, mainly composed by hydrophilic substances (Figure 4B). Given the broad nature of this classification system, many DESs might fit into multiple categories and it often creates confusion. According to the definition of DESs, the first two types better fit the ILs definition, since electrostatic interactions are predominant compared to H bonds. Types III and V include the majority of DESs studied in pharmaceuticals due to their advantages such as low toxicity, a wide range of possible combinations, non-reactivity with water, and biodegradability [29]. In this vein, the classification of choline bicarbonate and geranic acid at a 1:2 molar ratio (CAGE) has also been controversial. Geranic acid is a polyunsaturated fatty acid that is an octa-2,6-dienoic acid bearing two methyl substituents at positions three and seven. As stated by the authors that developed the CAGE compound, it fits the definition of an IL since it comprises largely ionic species, cholinium and geranate, and has a melting point below 100 °C. At the same time, CAGE is not a classical IL since it also contains neutral geranic acid [30]. NMR and molecular dynamic simulation studies revealed that there are complex interactions between the two species, making its classification difficult. The same authors refer to CAGE as ILs or even as type 3 DESs [31]. The same considerations can be made for choline glycolate (CGLY), identified by the authors themselves as an IL and DES [32] (Angsantikul et al., 2021), indicating that these species are somewhere in the middle of the two definitions [30].

In the last decade, it has been necessary to make a further distinction between DESs and natural deep eutectic solvents (NaDESs), which substantially match with class V. Natural eutectic systems can be considered biocompatible and biodegradable as well as environmentally safe. The term NaDESs first appeared in the study by Choi et al. in 2011 [33] to indicate DESs made up of substances of natural origin. The idea behind natural deep eutectic solvents (NaDESs) stems from the discovery of eutectic mixtures that naturally occur in microorganisms and plants. These mixtures serve as an alternative liquid phase, facilitating the synthesis of biological substances, preserving poorly soluble compounds, and protecting plants from cold temperatures. Therefore, NaDESs derive from metabolites that physiologically exist in various cells and organisms. The main constituents of NaDESs are organic acids, sugars, amino acids, and choline (Figure 4C). All of these substances are characterized by carboxyl, hydroxyl, or carbonyl groups capable of forming an intermolecular network of hydrogen bonds, responsible for the particular structure of these solvents [34].

### Therapeutic Deep Eutectic Solvents

Similar to the concept of API-ILs, therapeutic deep eutectic solvents (TheDESs) are eutectic mixtures in which at least one of the DES components is an API. Over the years, TheDESs have gained a certain notoriety as they offer various advantages compared to API-ILs, e.g., they are easier to prepare since in most cases they do not require the use of chemical reactions or additional solvents, but the simple physical mixture of the components [35]. For oral delivery, these liquid forms can be obtained by the combination of APIs with a wide variety of compounds (e.g., amino acids, vitamins, alcohols, or organic acids) or permeation enhancers (e.g., fatty acids). TheDESs can also be prepared by the selection of two different APIs, resulting in dual function liquid forms [36].

In forming TheDESs, selecting appropriate excipients is crucial because their physicochemical properties can significantly influence the creation of a eutectic system with the maximum melting point depression. Precisely, the melting point of the excipient should be similar to that of the API and the melting enthalpy of the excipient as low as possible. Additionally, the activity coefficient of both the API and the excipient should remain low across the entire composition range. However, the melting point depression as well as the position of the eutectic point in the solid–liquid equilibrium phase diagram should be adjusted with the therapeutic dose of the API to get maximum benefit [37]. Most TheDESs reported in literature exhibit melting temperatures near or below the human body’s temperature and usually envisage topical and oral administration. Initially, the majority of these liquid forms were designed for transdermal purposes and included permeation enhancers, such as terpenes and fatty acids [36].

## 4. Preparation Methods of ILs and DESs

**Figure 5:**
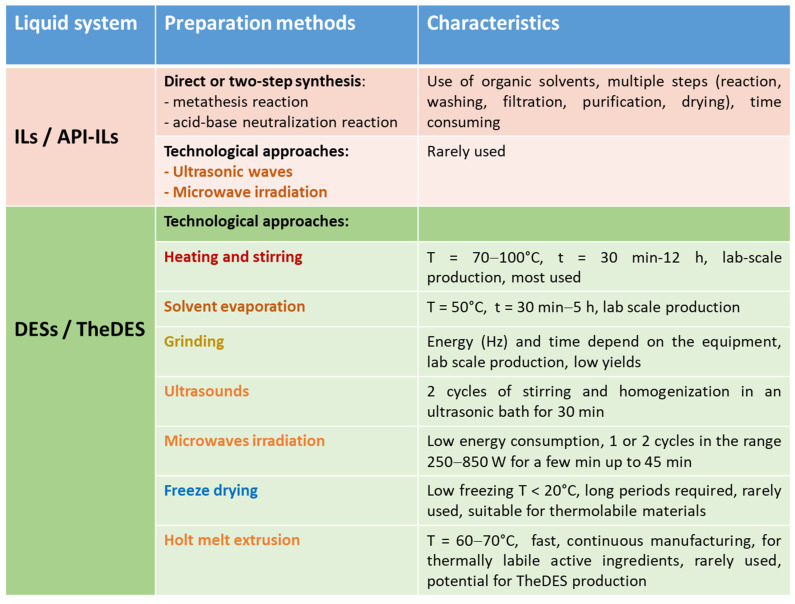
Summary of the different procedures used for the preparation of both IL and DES products and their application in oral DDSs.

This section details and evaluates the primary chemical reactions and fabrication techniques employed in the production of ILs and DESs, respectively, with a focus on their sustainability and scalability. Figure 5 provides an overview of the available production methods and their key characteristics, highlighting their use for the preparation of oral drug delivery systems (DDSs).

### 4.1. Synthesis of ILs and API-ILs

There are two primary methods for preparing Bio-ILs (namely, choline-based and amino acid-based ILs): the metathesis reaction and the acid–base neutralization reaction [11]. In a metathesis reaction, which means transposition, an anion exchange takes place between a quaternary ammonium salt and a metal salt, resulting in the formation of the thermodynamically most stable ion pairs. Conversely, in an acid–base neutralization reaction, an acid and a base directly combine to neutralize each other, forming the IL. These strategies can be time consuming and not very sustainable; for example, a metathesis reaction involves the exchange of ions between two chemical species in organic solvents such as methanol, leading to the formation of a new compound. The mixture has to be then filtered to remove the precipitate, leaving the ionic liquid behind. Further purification steps may be required to eliminate any remaining impurities. Recently, efforts have been made to produce ILs using more cost-effective and environmentally friendly methods, such as microwaves and sonication. Deetlefs and Seddon [38] were among the first to publish a microwave-assisted, solvent-free preparation of ILs based on nitrogen-containing heterocycles, achieving reasonably good yields. Similarly, Namboodiri and Varma [39] synthesized imidazolium halides and subsequently ILs using ultrasonication under solvent-free conditions, which were then used as reaction media.

Regarding API-ILs, they can be prepared through direct synthesis when the API is already in the desired salt form. In the metathesis reaction, a halide salt, hydroxide salt, or hydrocarbonate of the cation reacts with a free acid, metal, or ammonium salt of the anion. Particularly, when hydroxide salt or hydrocarbonate is available (e.g., choline bicarbonate), the reaction of the salt with the weakly acidic API containing the desired anion is straightforward and the by-product can be removed by washing, followed by filtration and drying under reduced pressure [19]. On the contrary, weakly basic API are commonly paired with decylsulfate, docusate, and oleate [7]. Metathesis reactions are commonly used to produce hydrophobic and water-insoluble API-ILs, making them ideal for lipid-based formulations (LBFs), such as SEDDS [40]. The formation of API-ILs typically occurs in a 1:1 molar ratio of the drug to the counterion. However, for APIs with multiple ionic groups, an excess of the counterion is used [41]. A two-step synthesis method is necessary when direct synthesis is unsuccessful. Various ILs, including commonly used tetrafluoroborate-, hexafluorophosphate-, and docusate-based API-ILs, are typically carried out via a two-step anion exchange reaction [42]. In the first step, halogenated alkanes and compounds containing target cations are quaternized to obtain halide salts containing target cations. In the second step, the API-ILs are obtained by ion exchange with acids or salts containing target anions. Also in this case, microwave irradiation or ultrasonic waves can be used to accelerate the reaction between the API and the ionic liquid precursor.

### 4.2. Fabrication of DESs and TheDES

DESs can be obtained through various strategies (namely heating, evaporating, grinding, and freeze-drying, recently summarized by Liu et al., [8]) that increase the entropy of the system, promoting the formation of eutectic mixtures. Here we focus on major pros and cons of the available methods and on techniques useful for potential large scale-production. The most frequently used method is heating, in which the components are combined and stirred at a specific temperature until a clear and uniform liquid is achieved. This method is easy to use and cost-effective, which explains its widespread adoption in laboratory studies. Various experiments have highlighted how the optimal temperature is below 100 °C (generally at 80–90 °C) [43]. The identification of correct heating temperature and duration of heating–stirring combination is critical for the successful preparation of DESs. Insufficient heating temperature or processing time can lead the formation of crystals [44]. Usually, a stirring time of 8–12 h is required.

An alternative method is the vacuum evaporation of the solvent, which was first reported by Dai et al. [45]. In this case, HBA and HBD are solubilized in a suitable solvent, generally water, after which the solvent is evaporated at a temperature around 50 °C using a rotary evaporator. The liquid obtained is placed in a desiccator with silica gel until a constant weight is achieved. The main drawbacks of this approach are the low yield and the long time required. For example, the preparation of CAGE consists in the addition of choline bicarbonate (80% solution) to purified geranic acid at a molar ratio of 1:2 stirring at 40 °C overnight. Water is removed by rotary evaporation at 60 °C for 2 h followed by drying in a vacuum oven for 48 h at 60 °C. The API candidate is then added to a specific volume of CAGE, followed by sonication or stirring for the time necessary for its solubilization [46] and used to formulate oral DDSs.

Freeze-drying is a scalable technological procedure that can allow the formation of DESs, but it is not particularly used for neat DESs preparation due to the long time required. This method starts with preparing separate aqueous solutions of each of the excipients taken in a particular molar ratio to each other, followed by their mixing. The resultant solution is frozen at a very low temperature and subsequently dried to obtain a clear viscous liquid. Zhang et al. [47] reported that for preparing DESs, heating is more appropriate for liquid acids, whereas freeze-drying is better suited for DESs containing thermally unstable components. When dealing with HBDs that have more carboxyl or hydroxyl groups, freeze-drying or rotary evaporation are more suitable methods than heating. Gutiérrez et al. [48] demonstrated that DESs can be obtained by the freeze-drying of aqueous solutions of the individual counterparts. They also showed that DESs could maintain unchanged the properties of incorporated preformed liposomes after the freeze-drying process, suggesting promising applications for this solid platform. It means that freeze-drying does not represent the first-choice production method but it can be approached for composite drug delivery systems containing thermo-sensitive active substances.

Neat grinding in a mortar or by high energy mills consists of mixing the DES-forming excipients in a particular molar ratio followed by grinding them until the formation of a homogeneous liquid. Florindo et al. [49] reported that grinding in a mortar with a pestle at room temperature was more effective than heating in reducing the viscosity of the resulting DESs. They also noted a higher degree of tunability when using different HBA:HBD molar ratios. Conversely, low yields, temperature increases using high energy processing, and limitations in treating hygroscopic materials make this fabrication method less effective than the vacuum evaporating method.

Hot melt extrusion (HME) enables the conversion of the starting powders into continuous extrudates through the synergistic action of heat and pressure. This method is advantageous for the large-scale production of DESs, particularly in continuous manufacturing processes. Crawford et al. [50] documented a production rate of around 6 kg/h for a type 3 DES (choline chloride–urea at a 1:2 molar ratio) using a twin-screw extruder, achieving space time yields that are four orders of magnitude higher than those obtained with the heating and stirring method. HME was also employed for the efficient production of a TheDES based on metronidazole and maleic acid for intravaginal delivery [51]. However, there are no studies in the literature regarding the use of DES in the extrusion process to obtain solid dosage forms for oral delivery.

Santana et al. [52] compared the physicochemical properties of three NaDES formulations (all based on organic acids, xylitol, and water at a 1:1:10 molar ratio) prepared by different methods: controlled heating and stirring, an ultrasound-assisted (UA) method, and a microwave-assisted (MA) method. They found that NaDESs exhibited similar properties, although UA and MA methods have proven to be faster and more efficient. Ultrasonic waves produce cavitation that is responsible for the interaction between HBA and HBD. Ultrasonication should last at least 30 min, or shorter time cycles can be repeated until complete liquid formation. Microwaves cause dipole rotation and the fast collision of the molecules resulting in the eutectic mixture in a very short time (depending on selected Watt and stirring rate). From the point of view of time and energy consumption, the MA method requires less time to form DES and consumes 650 times less energy than the heating method [53,54]. However, both UA and MA methods should be used carefully for thermally unstable starting materials as amino acids or sugars due to the difficulty in controlling the temperature.

Overall, the HME and MA methods, allowing reduced reaction times from hours to minutes, may be used for large-scale production, even though further studies are needed to optimize the manufacturing conditions, especially when using heat-sensitive materials. Therefore, from a sustainability perspective, the production of DESs can be considered a strategy to overcome some of the disadvantages of ILs, such as high melting points, high cost, and toxicity. The preparation of DESs, or, if applicable, TheDESs, is simple and cost-effective, as it does not involve multiple steps, separation and purification processes, or the use of toxic organic solvents. Moreover, the starting components are usually low-cost and mainly derived from natural, renewable sources [45]. On the other hand, the production of API-ILs is quite simple enabling directly a liquid formulation miscible with a lipid-based formulation (LBF) vehicle.

## 5. Toxicity of ILs and DESs

The idea of ILs as green chemicals arose from the fact that they are often non-volatile and non-flammable, unlike organic solvents. ILs have the advantage over organic solvents in being modulable by modifying the chemical structure of one or both members of the ion pair, allowing a certain control over both the physicochemical properties and the toxicological behavior. Over the years, the biological activity of ILs has been studied and several toxicity tests have been proposed to detect toxic effects in biological systems and also to evaluate damage at cellular and subcellular levels [55,56]. Similarly, with the growing use of several DESs, it has become essential to investigate their toxicity and biocompatibility. In addition, while most studies in the literature have focused on DESs and NaDESs, there have been relatively fewer investigations into TheDESs.

This section provides a summary and discussion of the toxicity of ILs and DESs intended for oral delivery, focusing on their impact on cells, and animal models.

### 5.1. Toxicity of ILs on Cell Lines and on Animal Models

The effect of a large number of ILs on cell viability was investigated using different cell lines including human colorectal adenocarcinoma HT-29 [57] and Caco-2 [58,59], and leukemia rat IPC-81 [60,61] cell lines. These studies showed that both cation and anion types can decrease cell viability. However, the lipophilicity of the cation was not the major factor in causing toxicity unless its side alkyl chain contained more than six carbon atoms, as observed in Vibrio fischeri. Longer chains significantly increased the cation’s lipophilicity and, consequently, its toxicity. Studies on membrane models using long-chain ILs (over ten carbon atoms) revealed that these ILs significantly alter membrane properties, causing rupture even at low concentrations (below 100 mM) and leading to membrane destruction at higher concentrations. In contrast, ILs with short alkyl chains (four carbon atoms) caused only minor membrane damage, even at concentrations of 500 mM. Overall, the data demonstrated that the ability to induce membrane breakage is strongly related to the length of the alkyl chains [62]. The likely mechanism, as described in the literature, involves the insertion of cation alkyl chains into the cell membrane, disrupting its integrity. This process occurs in several steps: a positively charged cation approaches a negatively charged phosphate group on the cell membrane, and then the cation’s alkyl chain interacts with the membrane’s acyl group (a long-chain alkyl group) through hydrophobic interactions, ultimately embedding itself into the cell membrane [63]. Few articles in the literature examine the toxicity of ILs in mice, confirming the significant impact of alkyl chain length on mammalian toxicity. A two-carbon chain showed minimal toxicity, even after multiple doses of 2000 mg/kg/day in mice. Conversely, a 10-carbon chain caused fetal death or teratogenic effects at just 100 mg/kg/day [64]. Overall, studies on ILs have demonstrated varying levels of IL toxicity across species, from bacteria to higher organisms. It has also been noted that toxicity primarily depends on the cationic part of ILs and the length of their side chains. Three possible strategies have been proposed to reduce the toxicity of ILs:

(a) The introduction of polar groups in the alkyl chains of cations (not necessarily at the end of the alkyl chain) can suppress their hydrophobic interactions to cell membrane molecules [60];
(b) ILs composed of bio-derived ions are expected to be less toxic, albeit some bio-derived ions such as choline tryptophanate are relatively toxic [65];
(c) Zwitterionization that allows the formation of less aggressive ILs against biological systems such as cells and tissues; they are less likely to accumulate in living organisms and can also be more easily degraded by microorganisms [66].

### 5.2. Toxicity of DESs on Cell Lines and on Animal Models

The broader application of DESs and NaDESs than ILs in the development of oral drug delivery systems has required greater attention regarding their toxicity on cells. The main studies conducted so far on the toxicity of eutectic mixtures in cell lines and animal models are summarized in Table 1. Specifically, the toxicity of DESs obtained from the combination of choline with four different HBDs were evaluated using different cell lines: OKF6 (normal oral keratinocytes), H413 (oral cancer), A375 (malignant melanoma), PC3 (prostate cancer), HepG2 (hepatocellular cancer), and MCF-7 (breast cancer) [67]. DESs at different dilutions were compared to individual components using the 3-(4,5-dimethylthiazol-2-yl)-2,5-diphenyltetrazolium bromide (MTT) assay and the results showed an appreciable toxicity of the tested DESs, with the exception of urea-based ones, which were found to be less toxic than the individual constituents. Analyzing several formulations comprising DESs and NaDESs [68] (Table 1), the cytotoxicity profiles in different cell lines such as MCF-7, human cervical cancer (HelaS3), human ovarian cancer (CaOV3), and mouse skin cancer (B16F10) cell lines revealed that all cell lines presented higher susceptibility to the choline–malonic acid NaDES, the only one without added water and with the highest viscosity value. Here, a correlation was first observed between the water content, viscosity, and cytotoxicity of NaDESs. Specifically, the results followed the trend: choline–malonic acid > choline–sucrose–water > choline–fructose–water > choline–glucose–water > choline–glycerol–water for HelaS3, MCF-7, and B16F10. In the case of CaOV3, the toxicity trend was similar, except from NaDES with fructose and glucose, where the toxic effect is reversed. Negligible toxicity was then observed with NaDESs without organic acids, as for instance those based on betaine with malic acid and proline or betaine–glucose [69]. Similar results were observed when combining choline chloride in a 1:1 molar ratio with various organic acids (citric, malic, and lactic) on HT-29, CaCo-2, MCF-7, and MRC-5 cell lines; in fact, when replacing organic acids with sugars as HBD, the cytotoxicity decreased (Popović et al., 2023) [70]. However, the dilution of each NaDES with a substantial amount of water (20% by weight) may have partially disrupted the hydrogen bond network, leading to an increase in the presence of free COOH groups. In 2024, Albertini et al. evaluated the biocompatibility of eutectogels (EGs) on human gingival fibroblasts (HGF) using the MTT assay. The EGs were derived from the gelation with the xanthan gum of NaDESs based on ChCl with citric or malic acids and from the gelation with HPMC of NaDESs based on lactic acid and glucose. Formulations containing citric or malic acid, despite having a pH similar to that of the lactic acid-based eutectogel, exhibited excellent cell viability after incubation, while significant toxicity was found only for the lactic acid-based EGs. This toxicity was likely related to the high amount of lactic acid and to the reduced extent of intramolecular hydrogen bonding in HPMC-based EGs compared to xanthan gum formulations resulting in a greater amount of free COOH groups [71]. The cytotoxicity of limonene-based TheDESs on the Caco-2 and HT-29 cell lines was tested [72]. All samples exhibited antiproliferative values but ibuprofen–limonene (molar ratio 4:1) was selected for further studies as it did not hamper the cell viability of normal colonic cells. It was observed that ibuprofen and limonene used in different molar ratios, gave different results: the higher the limonene content, the greater the cytotoxicity of the TheDES. This can be explained by considering that limonene itself possesses cytotoxic properties. Furthermore, TheDESs based on limonene with ibuprofen also lead to an enhancement of the anti-inflammatory activity of ibuprofen.

Regarding the in vivo toxicity studies (Table 1), the acute toxicity of a series of choline-based DESs in a murine model was performed and their LD50 was determined following a single oral administration [67]. Choline/urea at a 1:3 molar ratio caused immediate death in animals, whereas the 1:2 composition had a toxicity comparable to glycerol, ethylene glycol, and triethylene glycol. Blood biochemistry revealed an increase in transaminases, indicative of liver damage. The important conclusion of this study was that these choline/urea DESs were not completely toxicity-free. Based on this result, Jung et al. [73] performed an in vivo study using a DES based on choline and urea at a 1:2 molar ratio. Mice were acutely exposed to the eutectic mixture (1.5 g/kg) via oral administration and its effects were investigated in comparison with those of choline, urea, an aqueous mixture of choline and urea, and saline solution. Metabolomic analyses of the liver, kidney, and serum revealed alterations in the metabolism of glutathione, nicotinamide, taurine, pyruvate, lactate, and lysophosphatidylcholines, suggesting that DES administration induced oxidative stress. This condition was confirmed by biochemical assays and the study also revealed ammonia stress. Therefore, DESs containing urea should be used with caution because they may contain ammonia, which seems to be responsible for the toxic effects. Oral twice daily administration for 14 days of 3 mL of betaine–glycerol NaDES induced mortality in two rats [74]. In addition, it induced excessive water consumption, reduced dietary intake and weight loss, hepatomegaly, and plasma oxidative stress associated with high blood lipid levels. This work demonstrated the toxicity after the oral administration of the selected NaDESs under short-term conditions; however, the authors conclude that toxicity may be due to the high oral dose administered to rats. Acute toxicity studies, which involved orally administering doses of 1.5, 3, 4.5, and 6 g/kg of betaine–mandelic acid (1:1) NaDES to mice, indicated no significant toxicity symptoms up to 4.5 g/kg. However, at the highest dose, one death was observed. Consequently, the authors concluded that this NaDES is safe for clinical use, with an LD50 greater than 5 g/kg [75]. Furthermore, a recent study [76] compared the toxicity of DES (N,N-diethylethanolammonium chloride–triethylene glycol) and NaDESs (choline–fructose and choline–glucose) in various cell lines and in vivo using mice models. NaDESs were observed to be less toxic than DESs across all cell lines, while in vivo NaDESs showed greater toxicity than DESs. The explanation was attributed to the higher NaDES viscosity, which results in a reduced circulation of the mixture in mice, a higher threshold concentration, and ultimately liver failure.

To summarize, these studies show that some DESs exhibit non-negligible toxicity. Several critical factors have been recognized as influential in the cytotoxic effects of certain DESs in both in vitro and in vivo models. These factors include the choice of various HBA or HBD, the presence of organic acids as HBDs (which can alter pH), the molar ratios of the components, the water content and viscosity, the dilution levels of DESs used in tests, the potential for synergistic toxicity, and the inclusion of components that modify metabolic pathways.

## 6. Applications in Oral Drug Delivery

The use of ILs has rapidly advanced, opening new frontiers in healthcare, particularly in enhancing drug delivery. This section explores the most recent applications of both ILs and API-ILs in oral drug delivery, highlighting their roles as alternative solvents for poorly water-soluble APIs and other active compounds that are challenging to formulate orally. The high potential of DESs lies in their characteristics as nearly ideal solvents: economical, stable at room temperature, and versatile. However, their non-negligible toxicity has restricted their pharmaceutical applications. Consequently, the use of natural deep eutectic solvents (NaDESs) and therapeutic deep eutectic solvents (TheDESs) has gained prominence in oral drug delivery systems.

### 6.1. Solubility Enhancement

Evaluation of ILs: The use of ILs as alternative solvents was first introduced by Jaitely et al. [77] when they used imidazolium-based ILs to solubilize potassium penicillin V, dexamethasone, dehydroepiandrosterone, and progesterone. Subsequently, recent studies have shown that ILs can improve drug solubility by several orders of magnitude compared to water. For example, Caparica et al. [78] have selected two ILs based on choline and amino acids (choline–phenylalanine and choline–glutamate) to improve the solubility of two poorly soluble drugs such as ferulic acid and rutin. The two ILs showed significant increases in drug solubility, especially for rutin, which was increased 6-fold in presence of choline–phenylalanine. It has been observed that the solubility of the drugs in ILs is influenced by the presence of an alkyl side chain; in particular, the solubility decreases with the increase in the length of the alkyl chain of the cation [79], despite having been seen to cause greater toxicity. Furthermore, experimental and molecular dynamics modeling studies were performed to explore the solubilization mechanism in ILs. Dasari and Mallik [80] used molecular dynamics simulations to understand how the cardiovascular drug LASSBio-294 dissolves in ionic liquid (IL) aqueous solutions. They found that hydrogen bonding, π–π stacking, van der Waals interactions, and Coulombic forces play a role in the solubilization process. Additionally, nuclear magnetic resonance (NMR) experiments confirmed that hydrogen bonding between the drug and IL is the main factor driving its high solubility. [81,82]. Shahani et al. [83] discovered that the solubility of the antimalarial drug quinine significantly increased in ILs like choline acetate, choline glycinate, and threoninate. These ILs are characterized by shorter alkyl chains, polar properties, and smaller anions. Furthermore, API-ILs have demonstrated effectiveness in improving the solubility of specific APIs. For instance, cholinium nalidixate, cholinium niflumate, and sulfasalazine cholate have increased the solubility of their respective APIs by 5000, 56,000, and 4000 times compared to their raw forms [42]. On the other hand, due to their interfacial properties, long alkyl chain API-ILs able to self-assemble into micelles can provide a significant solubility enhancement of hydrophobic APIs. In addition, these compounds, also known as lipophilic salts, can be combined with LBF vehicles, such as SEEDS, to improve the solubility of basic drugs with a strong tendency to recrystallize in the pH environment of the intestine, such as cinnarizine [84], and thus enhancing their oral drug absorption [41]. ILs have also shown the capability to solubilize polymers. Considering oral application, a blend of high-amylose starch and microcrystalline cellulose was solubilized in an imidazolium acetate IL for use in gastric-floating drug delivery systems [85].

Evaluation of DESs: Shekaari et al. [86] used choline–glycerol and choline–ethylene glycol eutectic mixtures to increase the solubility of acetaminophen and lamotrigine. The DES based on choline and ethylene glycol was the best solvent for both drugs. Lomba and coworkers observed that sugar- and choline-based NaDESs were able to improve the solubility of furosemide (BCS class IV) but not that of caffeine (BCS class I) [87]. The solubility of anthofoxacin hydrochloride, a fluoroquinolone, was increased in a DES composed of choline–para-toluene sulfonic acid compared to ethanol–water and ethanol–acetonitrile mixtures [88], while a DES composed of choline–propylene glycol–water (molar ratio 1:1:1) was able to improve the solubility of salsalate, a BCS class II drug [89]. Mokhtarpour et al. [90] evaluated the solubility of naproxen in three DESs: choline–ethylene glycol, choline–urea, and choline–malonic acid. All compositions increased the solubility of the drug; in particular, DESs containing malonic acid as HBD allowed a solubility increase of more than 3300 times. The same authors carried out a similar study to increase the solubility of indomethacin, obtaining a solubility increase up to 17,000-fold [91]. Dai and colleagues [45] studied the potential of a series of NaDESs to increase the solubility of active natural products: rutin, quercetin, cinnamic acid, cartamine, 1,8-dihydroxyl anthraquinone, taxol, and ginkgolide B. The best result was obtained with quercetin in the NaDES xylitol–choline–water, which allowed an increase in solubility of over 400,000 times. The authors also noticed the strong effect of temperature on solubility in NaDESs; the higher the temperature, the greater the solubility. In a follow-up study, Dai et al. [92] investigated the influence of water addition to NaDESs containing quercetin and cartamine. The presence of water reduces hydrogen bonds in NaDESs; however, a small percentage was able to significantly reduce the viscosity without a significant modification of the solubility of the active molecules.

Two hypotheses on the mechanism involved in the drug solubility enhancement using DES have been suggested: the liquid crystal theory and the binding theory. In both cases, a fundamental role is played by the components of the DES in terms of structure and molar ratios. The liquid crystal theory explains how the eutectic mixture is internally organized in a polymeric matrix characterized by the presence of spaces in which the active molecule should be solubilized; changing the percentage of water involved changes in the size of the holes and therefore of this structure, leading to different results regarding solubility. Considering the binding theory, the solute becomes an integral part of the DES matrix due to the establishment of intermolecular interactions [93]. Further studies [94,95] showed that DESs were able to improve the solubility of several molecules including curcumin (a BCS class IV drug) and various BCS class II drugs such as indomethacin, aprepitant, celecoxib, flufenamic acid, naproxen, ibuprofen, probucol, and cinnarizine. Recently, Albertini et al. [96] investigated the molecular interactions between NaDESs and different APIs belonging to BCS class II (tolbutamide, nimesulide, domperidone, and cinnarizine). NaDESs based on choline, proline, organic acids, and sugars significantly improved the solubility of these APIs compared to buffer solutions at pH 1.2 and 6.8. Upon analyzing NaDES compounds with choline such as the HBA, it was observed that the solubility of smaller molecules increased when larger HBDs were used, while higher molecular weight APIs were better incorporated into the network formed by smaller HBDs. NMR experiments confirmed the formation of a robust supramolecular structure involving the protons of choline, organic acid, and water and highlighted the role of choline’s methyl groups in establishing hydrophobic interactions with the aliphatic or aromatic portions of the drugs, aligning with the binding theory. Therefore, API-NaDESs are able to form a complex structure at supramolecular level, emphasizing that drug solubility depends on a balanced network of hydrogen bonds and hydrophobic interactions. Finally, hard gelatin capsules filled with NaDESs, containing up to approximately 25% water by weight, maintained their integrity for up to six months. This indicates that all water molecules were involved in the hydrogen bond network, indicating that capsules could be a viable strategy for delivering drug-loaded NaDESs.

In addition to their use in formulating small molecules, NaDESs have also proven to be effective solvents for biological macromolecules. For instance, Dai et al. [45] showed that NaDESs increased the solubility of three high-molecular-weight natural products, namely, gluten, deoxyribonucleic acid, and starch, compared with water. For starch, the best solvent results in a glucose–choline–water 2:5:5 molar ratio, while the mixture of lactic acid, glucose and water in a 5:1:3 molar ratio increased the solubilities of both gluten and deoxyribonucleic, by factors of 88 and 34, respectively.

Regarding the behavior of TheDESs, Santos et al. [97] combined ethambutol and L-arginine with citric acid for the treatment of tuberculosis. The drug solubility in the TheDESs was significantly higher than that of water. Furthermore, TheDESs based on ethambutol with sucrose, glucose, and glycerol increased the solubility of the drug [98].

### 6.2. Design and Development of Oral Drug Delivery Systems

As previously reported, oral administration has advantages over injection and other delivery methods, making it the preferred route of administration whenever possible. Formulations based on ILs and DESs have been suggested as a strategy to solubilize both challenging small and macromolecular drugs/therapeutics for the development of various drug delivery systems. Table 2 and Table 3 present a summary of recent studies on the use of ILs and DESs in oral drug delivery. Table 2 focuses on ILs and DESs loaded with a drug, while Table 3 highlights those paired with a drug, referred to as API-ILs.

The first investigation on the oral delivery of DESs was performed by Faggian et al. [99] (Table 2). After a screening of 30 DESs, rutin was formulated in an NaDES composed of proline and glutamic acid, and a pharmacokinetic study was conducted in Balb/c mice, comparing the NaDES with a drug suspension in water. The test sample resulted in a two-fold increase in the maximum blood concentration (Cmax), while retarding the time corresponding to the maximum plasma concentration (Tmax) from 15 min with the aqueous suspension to an hour with the NaDES. Subsequently, a pharmacokinetic study involving mice examined three NaDESs containing berberine–proline–malic acid 1:2, proline–urea 2:1 and proline–malic acid–lactic acid–water 1:0.2:0.3:0.5 molar ratio. All tested NaDESs increased the area under the curve (AUC) compared with the aqueous suspension, with the proline–malic acid–lactic acid–water composition being the most effective [100]. In 2019, several NaDESs were tested as agents for improving the solubility, stability and delivery of curcumin [94]. The authors discovered that the solubility of curcumin in intestinal fluid, when dissolved in an NaDES composed of choline chloride and glycerol, was influenced by the fluid volume. Specifically, the concentration of curcumin decreased as the fluid volume increased, suggesting a significant breakdown of the supramolecular structure between curcumin and the NaDES. Further in vivo studies were conducted on rats, where the IL (also categorized as a type 3 DES) composed of choline and geranic acid (referred to as CAGE) was administered, loaded with the hydrophobic drug sorafenib tosylate, a powerful multikinase inhibitor. It was hypothesized that the drug–CAGE mixture forms a micellar/nanoemulsion system in the gastrointestinal environment which led to increased drug absorption [46]. In 2023, Chakraborty et al. [101] tested several DESs to increase the oral bioavailability of celecoxib, a BCS class II drug. The DES formed by choline and malonic acid (1:1 molar ratio) provided an approximately 10,000-fold improvement in drug solubility and was selected for in vitro drug release studies in simulated gastrointestinal fluids. The results showed an initial high supersaturation, which decreased by approximately two-fold after 2 h. Animal pharmacokinetic studies established a significant improvement in both Cmax and AUC and times reduction in tmax showing that DESs represent a suitable system for improving the solubility and dissolution rate of orally administered drugs.

Additionally, various biological therapeutics, including insulin, monoclonal antibodies, and immunoglobulin (IgG) have been successfully formulated in ILs/DESs for oral administration. Notably, CAGE-based IL formulations significantly enhanced the paracellular transport of insulin by protecting it from enzymatic degradation within enteric-coated capsules, thereby promoting interactions with the mucus layer in the gastrointestinal tract [30]. Furthermore, Peng et al. [103] developed CAGE gel patches (“ionogel” CAGE-patches) loaded with insulin to improve the localization of the ILs in the intestine and improve the therapeutic window. In vitro transport studies revealed more than a 30% increase in insulin transport across Caco-2 and HT29-MTX-E12 co-culture layers when compared to controls. In the potential clinical setting, the authors have hypothesized that patches would be loaded into enteric-coated capsules that dissolve and release patches into the small intestine. A different IL/DES compound based on choline bicarbonate and glycolic acid, precisely choline glycolate (CGLY), was demonstrated to significantly increase the absorption of a model IgG antibody in rats after injection to the jejunum [32]. Potential mechanisms for enhancing oral absorption include improving the apparent solubility of the bioactive compound, reducing the integrity of intestinal tight junctions to promote paracellular permeation, permeating through the mucosal barrier by decreasing mucosal viscosity, and inhibiting proteolytic enzymes [32]. Peng et al. [102] conducted a fascinating in vitro study examining the impact of various choline-based ionic liquids (ILs) as mucus-modulating agents on the diffusion rates of cationic dextran through the mucus barrier and intestinal epithelium. The study highlights the potential of these ILs to enhance the oral absorption of cationic molecules usually trapped by the intestinal mucus layer.

As shown in Table 2, most of the reported applications involve pharmacokinetic studies after administering to animals neat DESs with the drug solubilized in them. So far, there are few studies regarding the transformation of the API-DES/NaDES solutions into an actual oral drug delivery system. For instance, Mukesh et al. prepared an ion gel through the self-polymerization of a methacrylate polymer in an NaDES formed by choline chloride and fructose containing dissolved indomethacin [104]. The drug delivery was influenced by the ion gel solubility in the dissolution media. Liu et al. [75] investigated amorphous solid dispersions (ASDs) incorporating NaDESs. The study demonstrated that the synergistic effect of NaDES composed of betaine and mandelic acid, along with ASD based on PVP K30 as a polymeric carrier, maintained drug supersaturation, thereby facilitating absorption. This delivery system not only improved the apparent solubility of the drug, but also reduced its cytotoxicity, thereby enhancing its oral bioavailability. The same polymer (PVP K30) was combined with carnitine–ethylene glycol (1:4) DES up to 15% w/w, and used to dissolve a poorly water-soluble model drug to create a supersaturating system indicated as a polymer-embedded deep eutectic solvent (PEDES). This system aimed to achieve the “spring and parachute” drug release effect. According to Panbachi et al. [105], this liquid had the potential to be developed into a viable formulation, such as a capsule or potentially an amorphous solid. More recently, eutectogels obtained through the gelation of drug-loaded choline chloride-based NaDESs using xanthan gum and water have been developed as a new pediatric oral delivery system to enhance the bioavailability of benznidazole, a first-line drug used for the treatment of Chagas disease. In vitro studies evidenced drug supersaturation conditions following the dilution of eutectogels with simulated gastrointestinal fluids. Pharmacokinetic studies performed on rats revealed that the drug’s bioavailability increased 2.6-fold, with a similar increase observed both in blood and in cerebrospinal fluid. These results demonstrate that NaDESs could be simply transformed in effective drug delivery platforms suitable for oral administration to pediatric patients [71]. A different formulative approach was reported by Said et al. [106], who developed nanocarrier systems combined with a choline–glycerol DESs (Table 2) to improve poor drug bioavailability.

Very interesting results were also obtained synthetizing API-ILs systems, summarized in Table 3.

Zhang et al. [107] proposed an API-ILs based on sodium dodecyl sulfate and amitriptyline hydrochloride obtaining controlled drug release vesicles with higher drug loading content compared to conventional drug delivery systems. Instead, different API-IL compounds have been designed to enhance the bioavailability of poorly water-soluble small molecules. Williams and colleagues [108] investigated the use of hexyl-3-hexyloxycarbonylpyridinium dicyanamide to synthetize API-IL with danazol and itraconazole. Their solubility was increased 20-fold and over 500-fold, respectively, compared to their solubility in soybean oil, a commonly used lipid excipient. The oral administration of the self-emulsifying IL formulation containing danazol increased the bioavailability of the API up to 4.3 times compared to the respective crystalline drug and prolonged the plasma exposure to the API compared to the respective lipid-based formulation. Likewise, itraconazole, cinnarizine, and halofantrine were transformed into lipophilic ILs developing oral SEDDS (self-emulsifying drug delivery systems) to facilitate their incorporation into lipid-based formulations and assimilation into the lipid absorption pathway [84]. The pharmacokinetic evaluation upon the administration of SEDDS revealed higher drug plasma exposure for the API-IL formulations (2-fold for cinnarizine and 20-fold for itraconazole) in comparison with the SEDDS with the respective parent APIs. Furthermore, the use of API-IL formed by antimalarial lumefantrine and dioctyl sulfosuccinate sodium salt (sodium docusate) increased drug loading in LBFs compared to the free drug, resulting in up to 35-fold improvement in plasma exposure [109]. Similarly, the use of Favipiravir (FAV)-ILs not only improved the apparent drug solubility, but also enhanced the pharmacokinetic and pharmacodynamic properties compared to free FAV. Among several FAV-ILs tested, upon oral dosing in mice, the FAV-βalanine ethyl ester formulation showed an increase in the absolute bioavailability by 1.9-fold compared with the control FAV formulation [110]. The same authors investigated the conversion of methotrexate into a series of five ionic liquids (ILs), and in vivo pharmacokinetic studies revealed that the API-ILs enabled 4.6-fold improved oral bioavailability compared with the raw drug and enhanced the antitumor activity [111]. Recently, a BCS class IV drug, chlorpromazine, has been transformed into a lipophilic API-IL by a metathesis reaction using sodium docusate, loaded into an LBF vehicle and encapsulated in cellulose-based and methacrylate polymers to obtain a solid dosage form [40].

Overall, these studies highlight the great potential of ILs, especially of API-IL, to improve the oral delivery of poorly soluble drugs.

## 7. Final Considerations and Future Perspectives

Over the past decade, ILs, DESs, and their respective natural and therapeutic subcategories—Bio-ILs, API-ILs, NaDESs, and TheDESs—have emerged as cutting-edge solvents capable of enhancing the bioavailability of small chemical entities. In addition, they could pave the way for the development of new delivery systems for small- and high-molecular weight molecules, such as biopharmaceuticals.

Both ILs and DESs exhibit low volatility, a broad range of thermal stability, and high solvent capacity. However, DESs are generally easier to prepare, more sustainable, and less expensive than ILs. The most studied class of ILs is that based on imidazolium, but the significant toxicity and scarce biodegradability limit their use in pharmaceutical formulations. More recently, new classes of ILs based on naturally occurring compounds (Bio-ILs) have emerged, but further in-depth studies are needed to assess their toxicity and biocompatibility. Future research should also focus on developing new tools for screening anions and cations to achieve the formation of safer ILs with the desired chemical and biological properties or to accurately characterize and quantify IL impurities for pharmaceutical applications. In the authors’ opinion, the development of API-ILs could be a beneficial strategy enabling tunable active pharmaceutical ingredients. Understanding molecular interactions is essential for optimizing their design and effectiveness. Moreover, investigations regarding the interactions of API-ILs with GI fluids are still lacking. Transforming therapeutics into ILs could thus offer significant advantages, highlighting the importance of thorough research on their systemic effects and pharmacokinetics.

Similar to ILs, the toxicity of DESs was assessed using both in vitro studies and in vivo studies. Overall, literature findings show that some DESs exhibit toxicity, whereas their natural variant, NaDESs, seems to be safer. However, further research is required for a more comprehensive understanding. In general, several key factors have been identified as relevant for the cytotoxic effects of some DESs, including the presence of organic acids like HBDs (which alter pH), the molar ratios of the components, the occurrence of synergistic toxicity, and the inclusion of components that modify metabolic pathways. Increasing the use of in vivo models would be a crucial step for the future development and application of DESs in oral pharmaceutical formulations. In this regard, predictive methods for toxicity behavior and chronic and systemic toxicity studies are lacking.

Stability tests have typically been conducted over the short term; thus, for their future application, it is crucial to investigate the long-term stability of DES and IL-based formulations in greater depth. It should also be considered that there is still incomplete information regarding the supramolecular architecture of all of these systems including SAILs, API-ILs, and NaDESs.

In terms of administration routes, most studies focused on transdermal or topical ones, with the oral route being less explored and only recently gaining attention. Research should focus on the interactions of ILs and DESs not only with water and buffer solutions that simulate GI fluids but also with biorelevant media in both fasted and fed states. For instance, designing effective formulations requires a comprehensive understanding of NaDES behavior in biorelevant media, particularly how their dilution in these complex fluids affects drug apparent solubility and potential precipitation. In addition, the molecular mechanisms by which ILs and DESs increase the permeability of drugs should be further studied. Indeed, a thorough understanding of the mechanisms underlying the increase or decrease in bioavailability will facilitate a more rational application of these solvents in the drug delivery field. Finally, limited information is currently available about the interactions between DESs intended for oral delivery and the gut microbiome. DESs could potentially alter the composition of the gut microbiome, necessitating investigations into different types of DESs, their concentrations, and their effects on various microbial communities. However, it is important to consider that, as highlighted in toxicity concerns, DES formulations undergo significant dilution with GI fluids once ingested, which may weaken and alter the extent of these interactions. Therefore, investigation on this aspect is crucial for enabling viable oral formulations as well.

To conclude, this comprehensive literature review underscores the significant potential of these systems to improve the biopharmaceutical properties of APIs with low bioavailability. Despite the recent advancements in their properties and applications, further investigations on their supramolecular architecture, toxicity, and long-term stability and on their interaction with biological systems (GI fluids and mucosa) are still necessary. According to the authors, the development of innovative and efficient NaDES-based supersaturating oral drug delivery platforms and API-IL compounds may offer a promising strategy to obtain effective drug delivery systems suitable for oral administration.

## Figures and Tables

**Table 1:** Toxicity of DESs on cell lines and animal models.

	HBA	HBD	Molar Ratio HBA:HBD:H_2_O	Cell Line/ Animal Model	Results	Ref.
Toxicity on cell lines	Choline	Glycerol	1:3	OKF6, H413, A375, PC3, HepG2, MCF-7, and ICR mice	Choline-based DESs are not completely toxicity-free.	[67]
		Ethylene glycol				
		Triethylene glycol				
		Urea				
	Choline	Fructose	5:2:5	MCF-7, HelaS3, CaOV3, and B16F10	Toxicity observed only for choline–malonic acid NaDES.	[68]
		Glucose	5:2:5			
		Sucrose	4:1:4			
		Glycerol	1:2:1			
		Malonic acid	1:1			
	Choline	Oxalic acid	1:1	HEK293, HeLa cells, and MCF-7	Only NADESs that contained organic acid showed toxicity towards the test systems.	[69]
		Urea	1:2			
		Xylitol	5:2			
		Sorbitol	2:3			
	Betaine	Glucose	5:2			
		Malic acid–proline	1:1:1			
	Citric acid	Proline	1:1			
		Glucose–glycerol	1:1:1			
		Fructose–glycerol	1:1:1			
	Choline	Citric acid	1:1	HT-29, CaCo-2, MCF-7, and MRC-5	Before testing, each combination of HBA:HBD was diluted with 20% water (*w*/*w*). NaDESs with an organic acid component showed higher cytotoxicity than those containing sugars.	[70]
		Malic acid	1:1			
		Lactic acid	1:1			
		Ascorbic acid	2:1			
		Urea	1:1			
		Glycerol	1:1			
		Fructose	1:1			
		Sorbitol	1:1			
		1,3 propanediol	1:1			
		1,2 propanediol	1:1			
		1,3 butanediol	1:1			
		1,4 butanediol	1:1			
	Choline	Citric acid	2:1	HGF	NaDESs were transformed into eutectogels (EGs) using xanthan gum for choline-based NaDESs and HPMC for lactic acid-based DESs. No cytotoxic effect, except for lactic acid-based NaDESs.	[71]
			3:1			
		Malic acid	1:1			
	Lactic acid	Glucose	5:1			
	Capric acid	Limonene	1:1	Caco-2 and HT-29	TheDES composed of IBU:LIM (1:4) showed the lowest cytotoxicity.	[72]
	menthol		1:1			
	Ibuprofen	Limonene	1:4			
			1:8			
Toxicity on animal models	Choline	Urea	1:2	Mice	DES administration induced both oxidative and ammonia stress.	[73]
	Choline	Glycolic acid	2:1	Adult male Wistar rats	Safe for oral administration in rat models.	[32]
	Betaine	Glycerol	1:2	Rats	Toxicity observed following short-term oral administration (high dose given to the rats).	[74]
	Betaine	Mandelic acid	1:1	Mice	Acute toxicity studies using four different doses orally administered. Results indicated that the Bet–Man NaDES was well tolerated with acceptable toxicity (LD_50_ > 5 g/kg).	[75]
	N,N-diethyl ethanol ammonium chloride	Triethylene glycol	1:3	Mice	NaDES showed lower toxicity than DES in cell lines (HelaS3, A375, AGS, and WRL-68), but in vivo, the opposite effect was observed.	[76]
	Choline	Glucose	2:1			
		Fructose	2:1			

**Table 2:** Overview of studies on the use of ILs and DESs in oral drug delivery published in the last 10 years.

API	ILs/DESs Used	Oral Drug Delivery System	In Vitro/In Vivo Studies	Ref.
Rutin	Proline and glutamic acid at a 2:1 molar ratio	Neat NaDES	Mice received 10 mg of rutin by oral gavage as water suspension or NaDES. The oral administration of NaDES improved rutin bioavailability compared to the water suspension.	[99]
Berberine	Proline and malic Acid 2:1, proline and urea 2:1, proline–malic acid–lactic acid–water at a 1:0.2:0.3:0.5 molar ratio	Neat NaDES	Mice were administered 50 mg/kg of berberine via oral gavage, either as a water suspension or solubilized in the selected NaDESs. The best performance was achieved with proline–malic acid–lactic acid–water NaDES.	[100]
Curcumin	Choline chloride and glycerol at a 1:1 molar ratio	Neat NaDES	The solubilities of curcumin pre-dissolved in NaDES are 3.5 and 2 times higher than FaSSGF and FaSSIF, respectively. The dilution of NaDES with larger volumes of gastrointestinal fluids reduced curcumin solubility and delivery than small volumes.	[94]
Sorafenib tosylate (SRF)	Choline bicarbonate and geranic acid at a 1:2 molar ratio (CAGE)	Neat CAGE Spontaneous formation of IL nanocomplexes after dilution in the GI fluids.	CAGE was delivered to rats by oral gavage. The plasma concentration of SRF from CAGE was 2.2-fold higher than that in SRF suspension.	[46]
Celecoxib	Choline and malonic acid at a 1:1 molar ratio	Neat NaDES	Supersaturation occurred in in vitro dissolution studies (FaSSIF). Pharmacokinetic studies on rats established 2.76 times improvement in Cmax, 1.52 times reduction in tmax, and 1.81 times improvement in AUC.	[101]
IGg	Choline bicarbonate and glycolic acid, at a 2:1, 1:1, and 1:2 molar ratio (CGLY)	Neat CAGE	CGLY with a 2:1 molar ratio showed excellent cell compatibility, IgG integrity preservation, and the best transport of IgG antibody across Caco-2 cell monolayer via the paracellular route. Intrajejunal administration of IgG in CGLY 2:1 improved antibody absorption into intestinal membrane of rats.	[32]
Dextran	CGLY	Neat CGLY	In vitro evaluation of choline-based ILs for mucus modulation and transport use of a coculture of Caco-2 epithelial cells and HT29MTX-E12 mucus-secreting goblet cells in a Transwell model.	[102]
Insulin	CAGE	Insulin-CAGE encapsulated in size 9 enteric-coated capsules with Eudragit L100	The interactions between CAGE and Caco-2 monolayers were investigated in vitro. The efficacy of insulin-CAGE was determined in rats both after injection into the jejunum and after oral administration of capsules using an oral gavage. CAGE demonstrated a concentration-dependent effect both on cellular viability in Caco-2 cells and on insulin transport.	[30]
Insulin	CAGE	CAGE with dissolved insulin entrapped within a physically cross-linked PVA gel, forming a mucoadhesive ionogel patch (CAGE-patch) designed to adhere to the intestine	An in vitro assessment of CAGE-patches was conducted to determine their effectiveness in facilitating insulin transport using a Transwell model with a coculture of Caco-2 and HT29-MTX-E12 cells.	[103]
Indomethacin	Choline chloride and fructose at a 2:1 molar ratio	Ion gel obtained by the self-polymerization of a hydroxyethylmethacrylate polymer in the NaDES	The drug release from the ion gel in PBS buffer solutions at different pH levels was closely dependent on the pH–solubility relationship of the ion gel.	[104]
RA-XII	NaDES composed of betaine (Bet)–mandelic acid (Man) at a 1:1 molar ratio	ASD composed of PVP K30– Bet–Man–RA-XII at a 3:1:1 weight ratio prepared using the solvent evaporation method	An in vivo pharmacokinetic study on rats demonstrated that the oral bioavailability of the drug in NaDES and ASD was enhanced by 11.5-fold and 7.5-fold, respectively, compared to a dispersion of the raw drug.	[75]
Indomethacin	L-carnitine–ethylene glycol at a 1:4 molar ratio	Polymer-embedded deep eutectic solvents (PEDESs) using PVP K30	In vitro drug release studies demonstrate the “parachute effect” of the polymer–DES combination.	[105]
Benznidazole	NaDES composed of: Choline–citric acid–water at a 3:1:0.4 molar ratio and Choline–malic acid at a 1 molar ratio	Eutectogels to be administered orally in a stick pack. They were obtained by adding xanthan gum (1% *w*/*v*) and water (10% *w*/*w*) into the drug-loaded NaDES	An in vivo pharmacokinetic study demonstrated a significant increase in BNZ concentrations in the bloodstream and cerebrospinal fluid of rats compared to the raw drug.	[71]
Lornoxicam	Choline chloride–glycerin at a 1:2 molar ratio	Desosomes and desimicelles obtained combining DES with vesicular and micellar systems, respectively	In vitro release studies demonstrated a controlled release profile of the drug at pH 6.8–7.4, with desimicelles exhibiting a more sustained release compared to desosomes. Oral administration to rats showed an early and extended anti-inflammatory response compared to pure drug. Micelles performed better than vesicles.	[106]

**Table 3:** Overview of studies on the use of API-ILs in oral drug delivery published in the last 10 years.

API-ILs	Oral Drug Delivery System	In Vitro/In Vivo Studies	Ref.
Amitriptyline hydrochloride (AH) combined with anionic surfactant sodium dodecyl sulfate	Neat API-IL: self-assembled vesicles for controlled drug release	In vivo drug pharmacokinetics after oral administration in rabbits confirmed in vitro results having longer plasma exposure compared with free drug.	[107]
Danazol with different pyridimiun derivatives-based ILs	Neat API-IL: Danazol-containing self-emulsifying methylpyridinium IL	A 4.3-fold higher exposure than the crystalline drug and more controlled drug release compared with a lipid formulation in simulated gastrointestinal conditions.	[108]
HCl salts of itraconazole, cinnarizine, halofantrine with decylsulfate or octadecylsulfate ammonium salts or with sodium oleate	LBF: Itraconazole docusate or cinnarizine decylsulfate API-ILs were dissolved in long-chain lipid SEDDS at high concentration	SEEDS formulations were administered to rats by oral gavage. Significant bioavailability enhancement compared to the suspension formulations.	[84]
Lumefantrine-docusate	Different LBFs The Type IIIB LBFs resulted in the most effective solubilization and supersaturation in vitro	The Type IIIB LBFs formulations, delivered to rats by oral gavage, significantly out-performed Type II and Type IV formulations.	[109]
Favipiravir (FAV) with a series of IL-forming biocompatible cations, including amino acid ester, cholinium, and quaternary ammonium ions.	Neat API-ILs	FAV-ILs were administered to mice through oral gavage.	[110]
Methotrexate (MTX)-amino acid ester (proline ethyl ester)	Neat API-ILs	Oral administration to mice showed 6-fold higher oral bioavailability than MTX sodium.	[111]
Chlorpromazine docusate	Polymeric microparticles encapsulating the self-emulsifying API-IL containing Type IIIB LBF	Improved drug release profiles in pH 6.8 and FassiF from microparticles compared to the liquid formulations.	[40]

## Data Availability

Not applicable.
